# Questions on unusual Mimivirus-like structures observed in human cells

**DOI:** 10.12688/f1000research.11007.1

**Published:** 2017-03-14

**Authors:** Elena Angela Lusi, Dan Maloney, Federico Caicci, Paolo Guarascio

**Affiliations:** 1 St Vincent Health Care Group, University College of Dublin, Dublin 4, Ireland; 2 Bioinformatics Solutions Inc., Waterloo, ON, N2L 6J2, Canada; 3 Department of Biology, University of Padua, Padua, 35121, Italy; 4 Liver Unit, St Camillo Hospital of Rome, Rome, 00152, Italy

**Keywords:** Mimiviruses, human cell structure, histone H4, retroviral antigen, polydnaviruses

## Abstract

**Background: **Mimiviruses or giant viruses that infect amoebas have the ability to retain the Gram stain, which is usually used to colour bacteria. There is some evidence suggesting that Mimiviruses can also infect human cells. Guided by these premises, we performed a routine Gram stain on a variety of human specimens to see if we could detect the same Gram positive blue granules that identify Mimiviruses in the amoebas. **Methods: **We analysed 24 different human specimens (liver, brain, kidney, lymph node and ovary) using Gram stain histochemistry, electron microscopy immunogold, high resolution mass spectrometry and protein identification. **Results:** We detected in the human cells Gram positive granules that were distinct from bacteria. The fine blue granules displayed the same pattern of the Gram positive granules that diagnose Mimiviruses in the cytoplasm of the amoebas. Electron microscopy confirmed the presence of human Mimiviruses-like structures and mass spectrometry identified histone H4 peptides, which had the same footprints as giant viruses. However, some differences were noted: the Mimivirus-like structures identified in the human cells were ubiquitous and manifested a distinct mammalian retroviral antigenicity. **Conclusions:** Our main hypotheses are that the structures could be either giant viruses having a retroviral antigenicity or ancestral cellular components having a viral origin. However, other possible alternatives have been proposed to explain the nature and function of the newly identified structures.

## Introduction

There is evidence that terrestrial giant viruses can also infect mammals and a recent article published on Lancet Infectious Diseases describes the presence of giant viruses in human lymph nodes ^1– 3^. One of the chemical characteristics of giant viruses is their property to retain the Gram stain, which is usually used to colour bacteria ^4, 5^.

In fact, Mimiviruses (giant viruses) were initially mistaken for gram-positive bacteria infecting the cytoplasm of an amoeba, which was stuffed with blue Gram positive granules under an optical microscope. Only in 2003 did electron microscopy clarify that the fine blue granules present in the cytoplasm of the amoebas were actually giant viruses ^6^.

Guided by the premise that giant viruses can also infect humans, we decided to perform a routine Gram stain on different human specimens to see if we could detect the same blue granules that were detected in the amoeba when Mimiviruses were first identified.

Here we demonstrate, with the use of electron microscopy, mass spectrometry and histochemistry, that human cells have anatomical areas that manifest some of the biochemical and morphological properties also found in giant viruses. These structures are ubiquitously present in a variety of human tissues, including non-pathological tissues. Possible alternative explanations of the findings are discussed.

## Methods

### Characteristics of human samples

- 3 liver specimens with haemochromatosis and non-alcoholic steatohepatitis
- 1 liver specimen with cryptogenic cirrhosis (unexplained)
- 7 liver specimens with chronic hepatitis B
- 2 liver specimens with chronic hepatitis C
- 3 liver specimens with non-specific minimal histological lesions
- 2 liver specimens with no lesions
- 2 liver specimens with primary biliary cirrhosis
- 1 liver specimen from a patient with Crohn’s disease
- 1 kidney specimen
- 1 brain specimen
- 1 ovary specimen

The Institutional Review Board of St Camillo Hospital of Rome approved the use of stored tissues for electron microscopy and proteomics investigations in accordance with the Helsinki Declaration of 2002 (approval number, 56/2015). Informed consent were obtained in writing from all patients prior to tissues biopsy procedure, which encompassed processing of the clinical data and the use of tissues for investigation and research. The present study fits within the terms of the obtained consent.

### Gram positive staining of human specimens

Gram staining of human specimens was performed using a Gram Yellow Stain Kit (Artisan from Dako), following the standard protocol for paraffin specimens, according to the manufacturer’s instructions. Positive controls were formalin fixed human tissues with bacteria. Before staining, slides were heated at 80°C for 45 minutes.

### Electron microscopy

Electron microscopy analysis of the human biopsies was conducted at the University of Naples Federico II, CISME Division and the University of Padua, Department of Biology. The two different operators were blinded to each other's. The samples were fixed with fixative (4% paraformaldehyde in PBS buffer solution), dehydrated and embedded in LR White Resin followed by polymerization at 58°C. Ultrathin sections (100 nm) were placed on Formvar-coated nickel grids (Maxtaform Grids; M200-Ni) and used the next day for immunogold labelling. For immunostaining reaction, the post-embedding immunogold method was applied. Nickel grids were immersed in 1% citraconic anhydride solution (Sigma) at 90°C for 30’. Subsequently, the sections were first treated with blocking solution (1% BSA, 0.1% Tween 20, PBS 1x), then incubated with primary mouse monoclonal antibody identifying common retroviral antigen among mammalian retrovirus (sc-65623; Santa Cruz Biotecnology; IgG _1_ provided at 100 µg/ml) diluted 1:50 for 1 hour at 37°C. Antibody binding was detected using a secondary goat anti-mouse IgG antibody at room temperature for 1 hour (British BioCell International; EM.GAM15EM), diluted to 1:100 and coupled to gold particles (15nm; British BioCell International). Sections were analyzed using an FEI Tecnai G2 transmission electron microscope operating at 100 kV. The images were acquired with TIA Fei software Cam 4.7SP3 ( https://www.fei.com/service-support/) and collected and typeset in Corel Draw X3 ( http://www.coreldraw.com/en/pages/coreldraw-x3/). Controls were performed by omitting the primary antibody, which resulted in absence of cross-reactivity.

### Sample preparation for proteomics two dimensional gel electrophoresis

Human samples were ground with liquid nitrogen. Six volume sample preparation buffer (9M urea, 2% ampholytes and 70 mM DTT) were added to the frozen powder, followed by three frozen/thaw cycles (liquid nitrogen −196°C/30°C). After incubation for 30 min at room temperature and centrifugation for 45 min at 15000xg the supernatant was removed and frozen in new tubes at -80°C. FFPE slices were treated with 0.5 ml Heptan for 1h at room temperature. Subsequently, 25µl methanol were added and mixed for 25min. After centrifugation (5min, 13200xg) the pellet was air dried and 100µl lysis-buffer (250 mM Tris pH 9.5; 2% SDS) were added. The sample was boiled for 2h, centrifuged (30min, 13200xg, room temperature) and the supernatant was used for SDS-PAGE.

### Two dimensional gel electrophoresis

Two dimensional gel electrophoresis (2DE) was performed according to standard 2DE techniques. Briefly, 50 µg of protein was applied to vertical rod gels (9M urea, 4 % acrylamide, 0.3 % PDA, 5 % glycerol, 0.06% TEMED and 2 % carrier ampholytes [pH 2–11], 0.02% APS) for isoelectric focusing at 1820 Vh in the first dimension. After focusing, the IEF gels were incubated in equilibration buffer, containing 125 mM trisphosphate (pH 6.8), 40% glycerol, 65 mM DTT, and 3% SDS for 10 minutes and subsequently frozen at -80°C. The second dimension SDS-PAGE gels (7x8x0.1cm) were prepared, containing 375 mM Tris-HCl buffer (pH 8.8), 12% acrylamide, 0.2% bisacrylamide, 0.1% SDS and 0.03% TEMED. After thawing, the equilibrated IEF gels were immediately applied to SDS-PAGE gels. Electrophoresis was performed using 150 V for 75 min until the front reached the end of the gel. After 2DE separation, the gels were stained with FireSilver (Proteome Factory; PS2001).

The 2DE gels used for comparison analysis were digitized at a resolution of 150 dpi using a PowerLook 2100XL scanner with transparency adapter.

### Western blotting

For western blot applications, two identical gels were run. One 2DE gels was stained with FireSilver or Coomassie for preparative applications and the other gel was used for western blotting to detect the proteins by immunostaining. Blotting of 2DE gels was performed using an Immobilon-P membrane (PVDF; pore size 0.45 mm; Millipore) and a Trans-Blot SD Semi-Dry Transfer Cell (BioRad) at a constant current 5 V overnight at 4°C using a blotting buffer consisting of 25 mM Tris–HCl, 192 mM glycine, 0.1% SDS (pH 8.3) and 20% methanol. For immunodetection of proteins, membranes were washed in TBST (20 mM Tris–HCl [pH 7.5]; 154 mM NaCl, 0.1% Tween-20) and blocked in TBST containing 2% (w/v) BSA for 2 h. Membranes were incubated with the primary antibody (sc-65623; Santa Cruz Biotecnology; IgG _1_) diluted 1:1000 for 2DE blot and 1:50 for 1D-blots in TBST containing 1% (w/v) BSA overnight and then incubated with anti-mouse IgG (Fc specific–peroxidase antibody produced in goat; A0168; Sigma; diluted to 1:2000 in TBST containing 1% (w/v) BSA) for 1 h at room temperature. Finally, the bound antibody was detected by incubating with Luminol for 1s-20min (Roth). The membrane was washed in TBST (5 times for 10 min) between all incubation steps.

### Trypsin-in-gel-digestion / nanoLC-ESI-MS/MS

Protein identification was performed using nano LC-ESI-MS/MS. The MS system consisted of an Agilent 1100 nanoLC system (Agilent), PicoTip electrospray emitter (New Objective) and an Orbitrap XL mass spectrometer (Thermo-Fisher). Protein spots from the membranes were in-gel digested by trypsin (Promega) (with and without citraconic anhydride treatment) and applied to nanoLC-ESI-MS/MS. Peptides were trapped and desalted on the enrichment column (Zorbax SB C18; 0.3x5 mm; Agilent) for five minutes using 2.5% acetonitrile/0.5% formic acid as eluent, then peptides were separated on a Zorbax 300 SB C18 column (75µmx150mm; Agilent) using an acetonitrile/0.1% formic acid gradient from 5 to 35% acetonitril within 40 minutes. MS/MS spectra were recorded data-dependently by the mass spectrometer, according to manufacturer's recommendations.

### Peptide synthesis

Synthetic peptide KTVTSMDIVYALK was synthesized by solid-phase technique using a multiple peptides synthesizer (SyroII; MultiSynTech GmbH) on a pre-loaded Wang resin (Novabiochem) (100–200 mesh) with Fmoc- *N*ε- *tert*-butyloxycarbonyl- l-lysine (Novabiochem). The fluoren-9-ylmethoxycarbonyl strategy was used throughout the peptide chain assembly, utilizing O-(7-azabenzotriazol-1-yl)-N,N,N′,N′-tetramethyluronium hexafluorophosphate (HATU) as a coupling reagent. Cleavage of the peptides was performed by incubating the peptidyl resins with trifluoroacetic acid/H2O/triisopropylsilane (95/2.5/2.5%) for 2.5 h at 0°C. Crude peptide was purified by reverse phase HPLC on a preparative column (Prep Nova-Pak; HR C18). Molecular masses of the peptide were confirmed by mass spectroscopy on a MALDI TOF-TOF using an Applied Biosystems 4800 mass spectrometer.

### PEAKS and peptide identification

Immunoblot positive bands from frozen and FFPE tissues, were analyzed by mass spectrometry (nano LC-ESI-MS/MS), using a Thermo Orbitrap XL with CID fragmentation.

A database search was performed first against human proteins contained in UniProtKB/TrEMBL ( http://www.ebi.ac.uk/uniprot) and virus proteins contained in UniProtKB/TrEMBL separately. After that, to reduce the risk of false positive results, the search was made against a combined human and viral database within a 1% false discovery rate. The search parameters were: 20 ppm precursor error tolerance, 0.6 Da fragment error tolerance, trypsin allowing non-specific cleavage at 1 end and a maximum of 3 missed cleaves, carbamidomethylation set as a fixed ptm, acetylation(k), oxidation (M), deamidation(NQ), formylation (K, Nterm), phosphorylation (STY) set as variable modifications.

The raw files were also processed through PEAKS Studio 8.0 (Bioinformatics Solutions Inc.) *de novo* and PEAKS DB modules. The parent mass error tolerance was set to 3 ppm, the fragment mass error tolerance was 0.6 Da. Carbamidomethylation of cysteine was set as a fixed modification and oxidation was set as a variable modification. The enzyme rules specified were trypsin, allowing non-specific cleavage at one end maximum and a maximum of three missed cleavages per peptide. The database searched was trEMBL (version is 2016_09). Only human and polydnaviridae proteins were searched, 1109386 protein sequences were searched along with a decoy database containing an equal number of proteins.

## Results

### Gram positive staining

We Gram stained 21 different types of human liver specimens. We initially chose the liver, since this organ is the bio-chemical processing centre and the cross road of microbial invasions of the human body.

**Figure 1.**
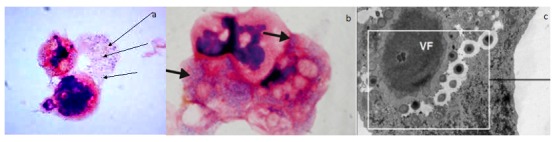
Premise picture. This picture illustrates Mimiviruses in the amoebas when first detected by the French authors ^6^. Viral particles appeared as Gram-positive fine blue granules (black arrows) resembling bacterial cocci, from which the name Mimiviruses, was derived, i.e. Mimicking microbes. The blue gram positive granules in the cytoplasm of the amoeba ( **A** and **B**) proved to be Mimiviruses and not bacteria when viewed using electron microscopy ( **C**). Permission to use this picture was kindly provided by Prof La Scola Bernard.

Gram positive blue granules were diffusely and ubiquitously expressed in all tested human liver samples, including unaffected liver samples with no histological lesions. These blue granules were absolutely distinct from common pigments, such as lipofuscin, and different from gram positive bacilli that were used as controls. The granules had a typically fine granular aspect, similarly to the one present in the amoebas infected by Mimiviruses, as reported by the French authors ^6^. Figure 1 (premise picture) illustrates Gram positive granules that are Mimiviruses infecting amoebas. The permission to use this image was kindly provided by Prof Bernard La Scola.

**Figure 2.**
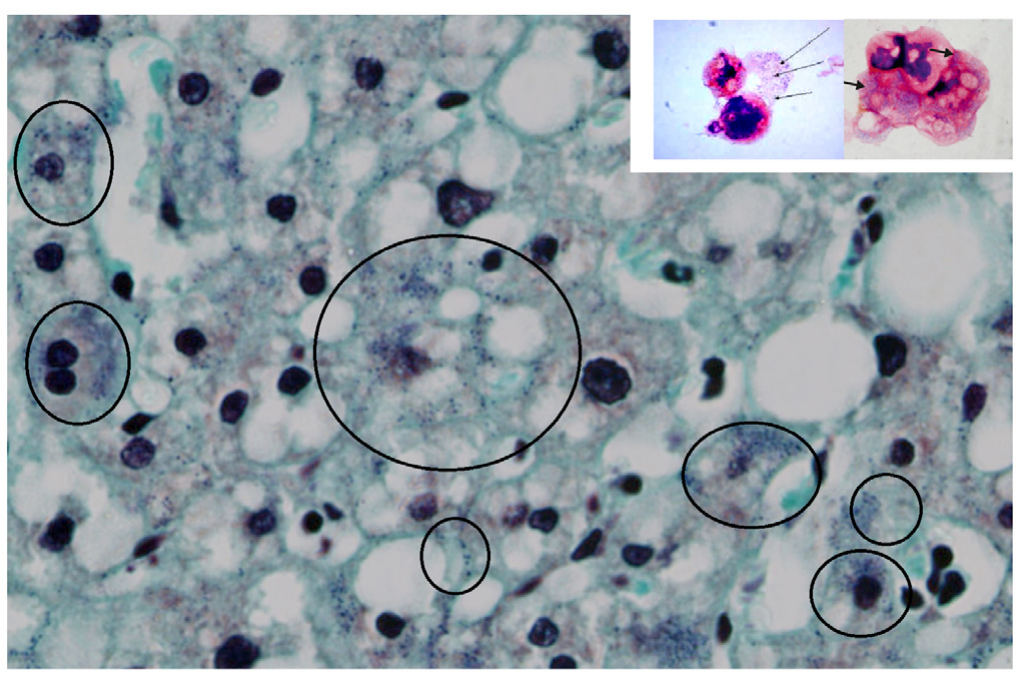
Human liver histochemistry. Gram staining of a human liver (magnification, ×80). After the Gram stain, human liver cells displayed fine blue granules that, for didactic reasons, are enclosed in the black circles, but they can be seen scattered in the parenchyma. Note the similarities between the amoebal Mimiviruses appearing as blue granules (small picture frame and Figure 1) with the human blue granules. In the human cells, the gram positive granules appear as fine granules and *are distinct* from bacteria and other pigment, like lipofucsin, that is also present (brown colour).

In the human liver cells, this fine blue granularity was detected in the cytoplasm and nuclei ( Figure 2).

**Figure 3.**
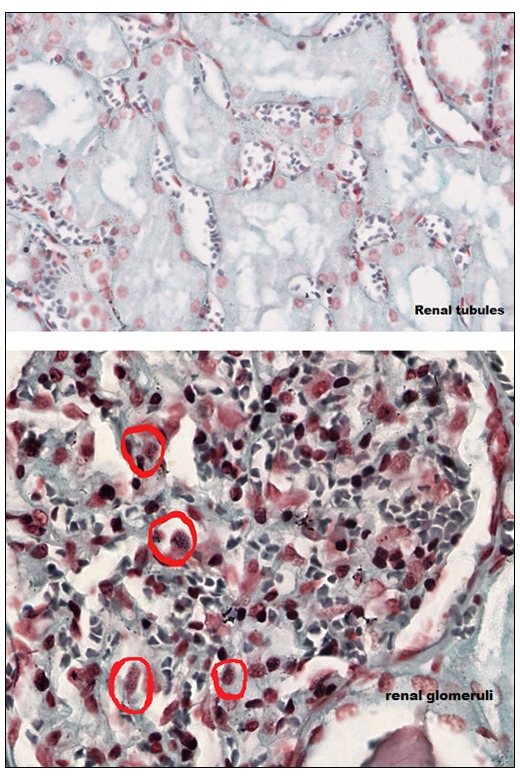
Human kidney histochemistry. Human kidney Gram stain. Absence of Gram positive granules in the renal tubules (magnification, ×43.3). Gram positive granules were only present on the glomeruli, red circles (×55).

**Figure 4.**
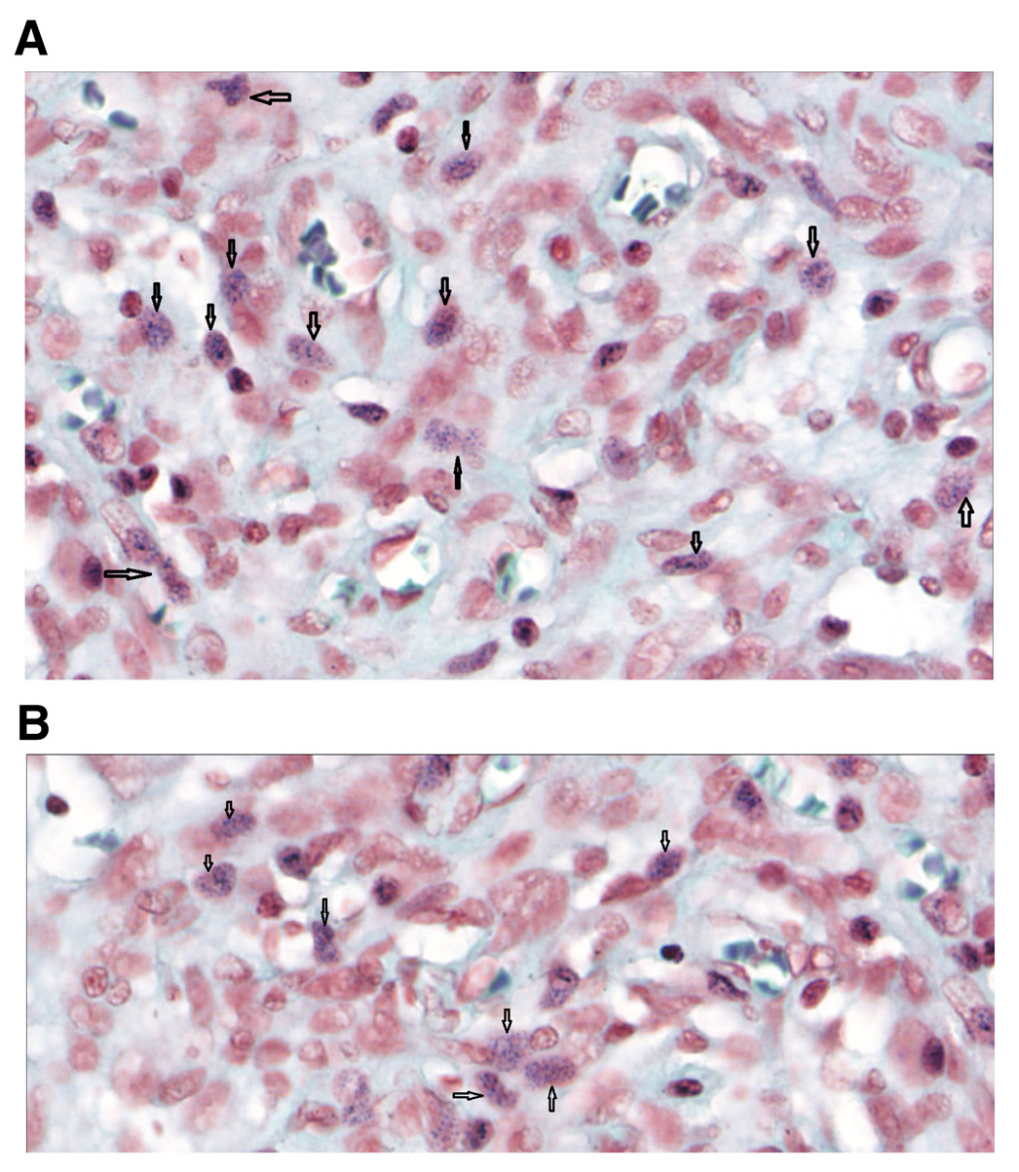
Human brain histochemistry. ( **A** and **B**) Human brain Gram stain (magnification, ×80). Gram positive granules were also present in the brain. Black arrows indicate the intracellular blue granules.

**Figure 5.**
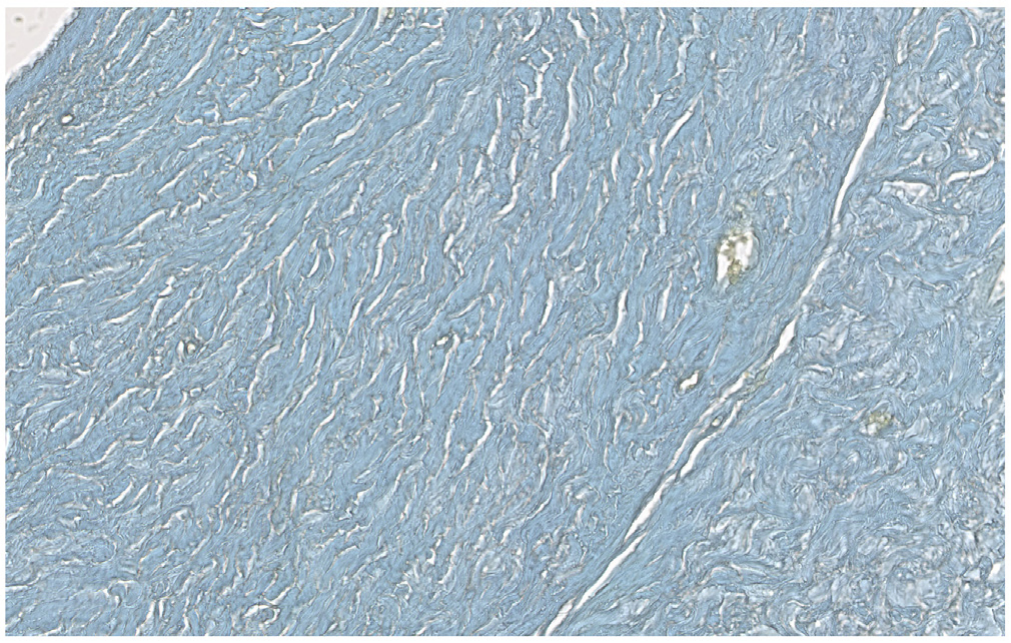
Human ovary histochemistry. Absence of Gram positive granules in the ovary (×20).

**Figure 6.**
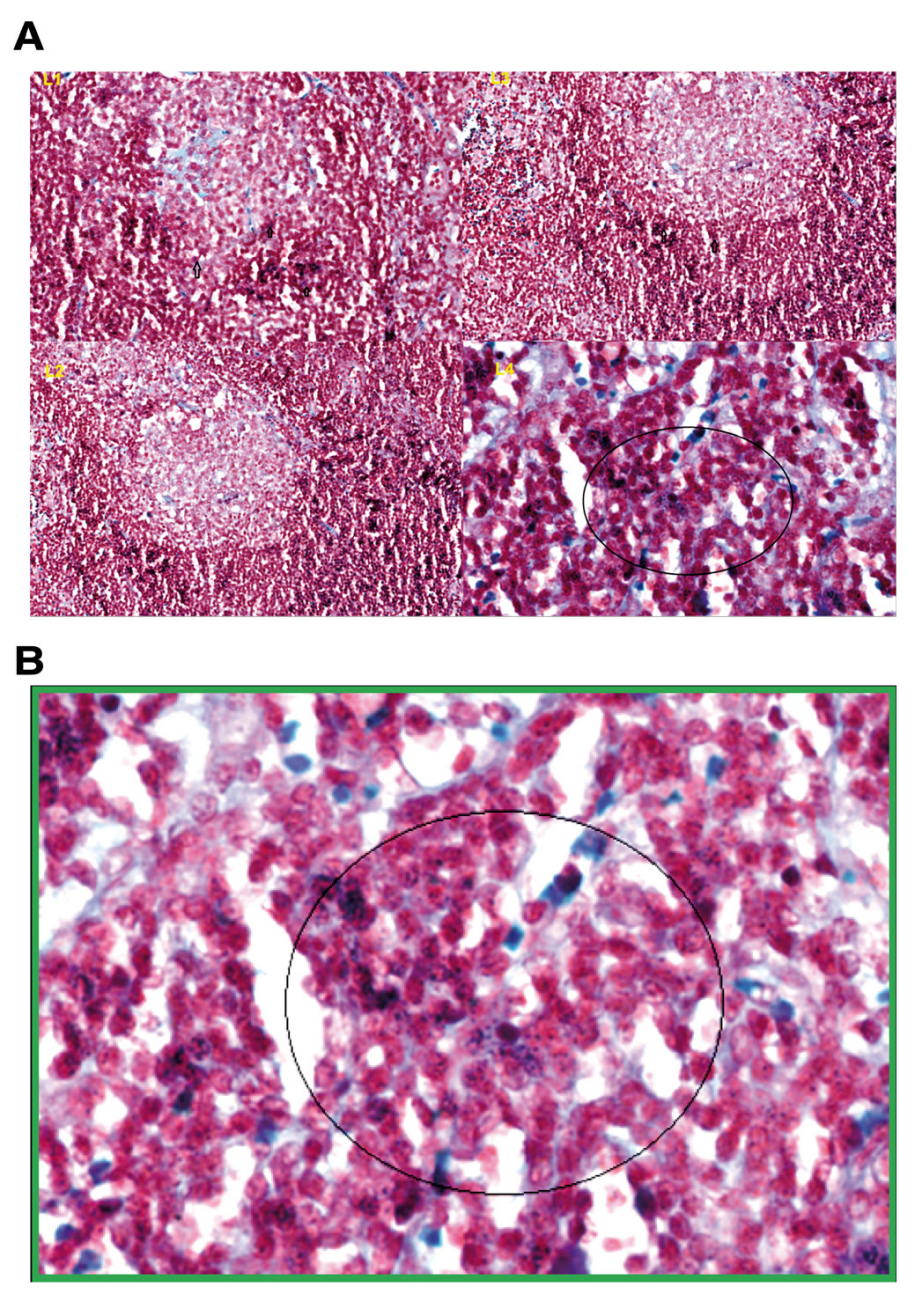
Human lymph node histochemistry. ( **A**) Gram positive granules were absent in the germinal centres L1, L2, L2. Gram positive granules were specifically detected in the paracortex, outside the germinal centres, L4. ( **B**) The picture with the green frame is L4 at higher magnification (×80) with blue granules in the circle.

To further verify the ubiquitous presence of these typical blue granules in the human cells, we also Gram stained human brain, ovary, lymph node and kidney tissues. Granules could be detected in the kidney glomeruli, but not in the renal tubules ( Figure 3). The brain was intensely stained, showing a diffuse granular pattern ( Figures 4A and B). The ovary did not display the gram positive granules ( Figure 5). In the lymph node, Gram positive granules were absent from the germinal centres and only appeared in the paracortex ( Figures 6A and B).

### Electron microscopy

**Figure 7.**
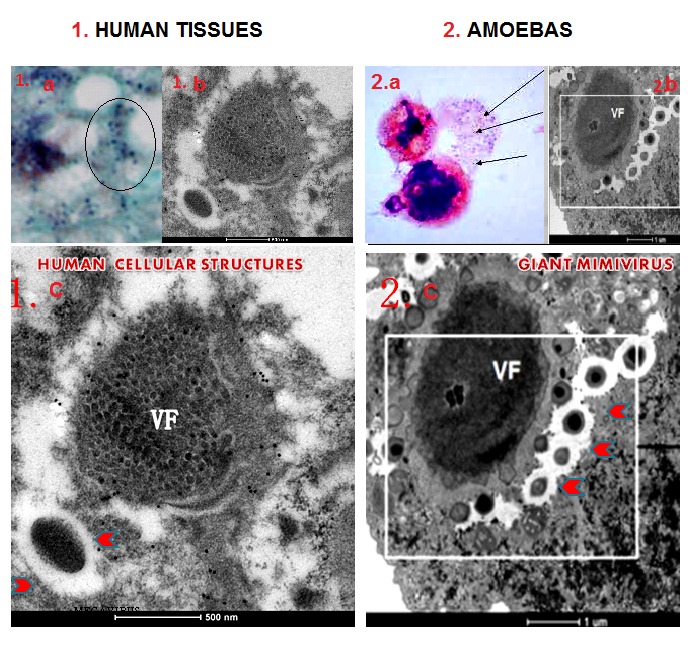
Representative micrograph of electron microscopy - comparative morphology. Electron microscopy (EM) of human liver tissues with the gram positive granules (black circle 1a) displayed Mimivirus-like structures at EM (1b). Similar gram positive blue granules in the amoeba (2a) are Mimiviruses (2b). Comparative morphological analysis revealed striking similarities between the human cellular structures (1c) with amoebas giant Mimiviruses (2c). Comparison of the two viral factories (VF) and the giant particles (red arrows) in the larger EM micrographs in section 1 and 2 show similarities between the two.

Subsequent electron microscopy (EM) analyses of the Gram positive human tissues confirmed the presence of cellular structures resembling Mimiviruses ( Figure 7). This was exactly the same case of the French authors when they proved that the fine blue granules in the amoeba were actually Mimiviruses ^6^. To enhance the resolution detection of the EM, we used a particular antigen retrieval solution with citraconic anhydride and heat ^7, 8^. EM analyses were conducted in two different international centres and 300 micrographs were scrutinized by operators who performed a blinded reading and were also blind to each other. The immunogold labelling assays revealed also a retroviral antigenicity associated to the structures when a mammalian anti-retroviral gag-p27 MoAb, recognizing common epitopes among several mammalian retroviruses, was tested.

### Mass spectrometry

Mass spectrometry (nano LC-ESI-MS/MS) and protein identification using PEAKS 7.5 software revealed the presence within the structures of human proteins, including conventional human histone proteins that co-existed with a histone H4 peptide **KTVT *S*MD *IVY*ALK.** This manifested a distinct viral footprint of giant polydnaviruses that did not match any human sequence. In fact, the human and many other eukaryotes display in correspondence of their C-terminus histone-H4 tail a typical and extremely conserved sequence **KTVT *A*MD *VVY*ALK,** with a I -> V replacement and human histone H4 variants that have never been described ^9, 10^.

To rule out false positive identifications when searching just with the virus database, we combined all identified proteins in the virus database and all identified proteins in the human database into one FASTA file ( Supplemental File 1). The raw files were processed through PEAKS Studio 8.0, *de novo* and PEAKS DB modules.

When analysing the biological samples, the peptide **KTVT SMD *IVY*ALK** was identified confidently in two replicates at similar retention times: 23.05 minutes in replicate one and 23.37 minutes in replicate two.

**Figure 8.**
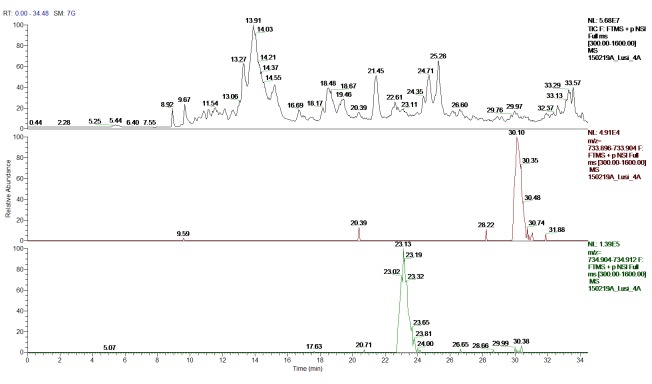
Peptide identification - ion chromatograms. Top-bottom: Total ion chromatogram (TIC); extracted ion chromatogram (EIC) DB-hit; EIC H4 histone variant.

To validate our results, a synthetic peptide with the same sequence as our candidate peptide **KTVT *S*MD *IVY*ALK** was produced at the CRIBI peptide facility, University of Padua. A significant number of high intensity b and y ions matched the synthetic peptide spectrum. In particular, the b and y ion series from ***IVY*** (the part of the sequence that differs from the human protein), were prevalent in both spectra. We also performed a narrow scan in the mass range 730–740 m/z; MSMS for center mass 734.40 m/z (2 ^nd^ isotope of 733.9 m/z; z=2); MSMS for center mass 744.40 m/z (2 ^nd^ isotope of 734.9 m/z; z=2). The canonical human histone H4 and the ***IVY*** histone H4 variant were both present at m/z= 734.907; z=2. A summary of the proteomics assays are reported in Figure 8– Figure 12 and in Supplemental File 2.

**Figure 9.**
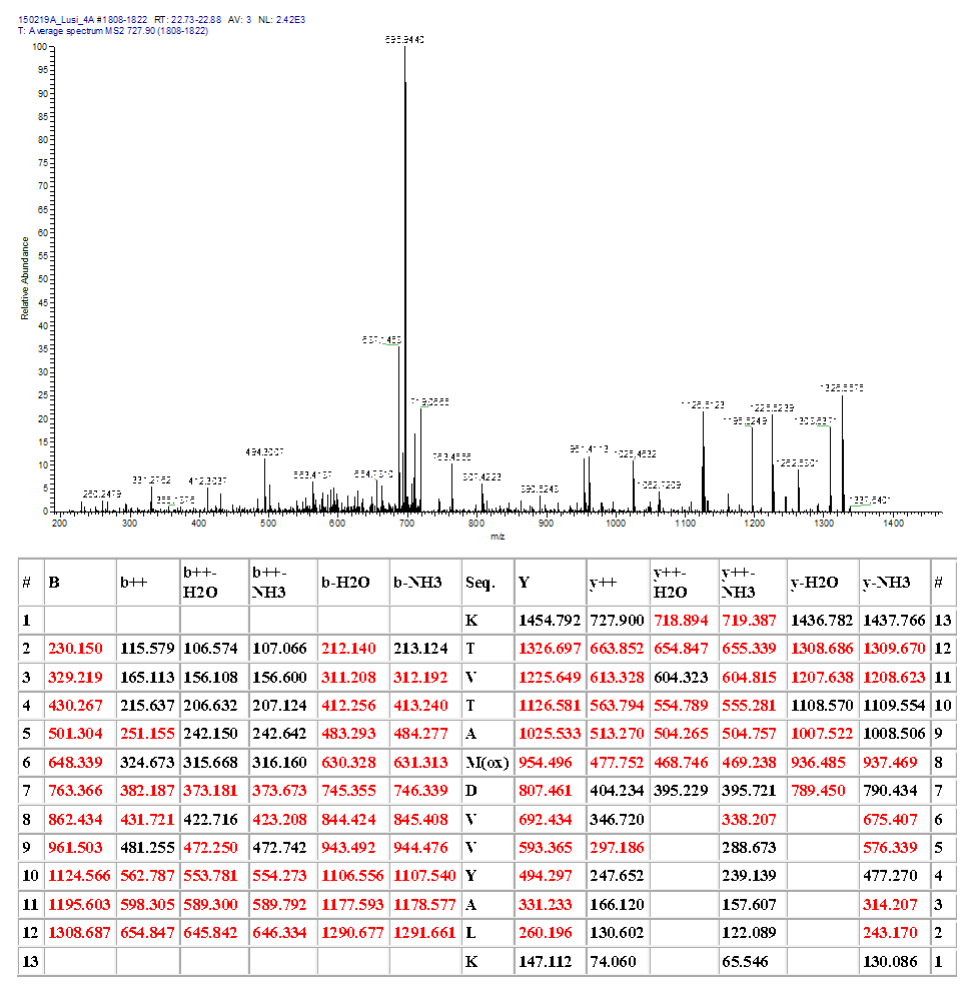
Mass spectrometry fragmentation pattern of the canonical human histone H4. Fragmentation table at t m/z= 734.907; z=2.

**Figure 10.**
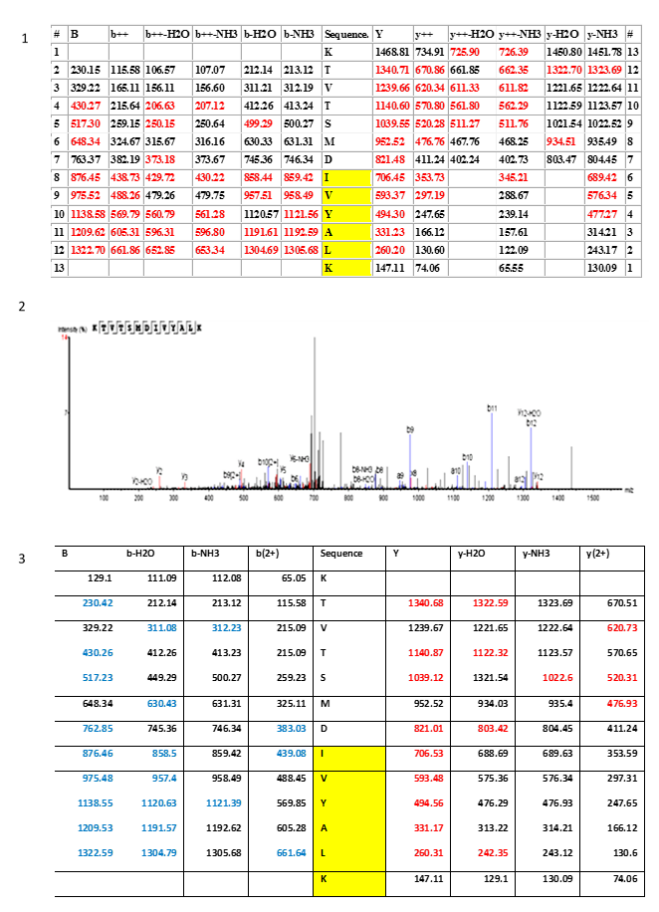
Mass spectrometry fragmentation patterns of the histone H4 variant (IVY). Section 1 and section 2 illustrate the fragmentation table and the spectrum (PEAKS software) of the ancestral variant of the histone H4 peptide KTVSMDIVYALK, respectively. Section 3 is the fragmentation table of the synthetic peptide that was synthesized and used to validate the KTVSMDIVYALK identification. Fragment ions that matched the spectrum in both the biological and synthetic spectrum are highlighted in colour. Red = xyz ions; blue = abc ions. The yellow region IVYALK indicates the histone H4 variant that was detected, along with the conventional histone H4, in the human cells. The same pattern IVY is also present in the histone H4 biology of polydnaviruses.

**Figure 11.**
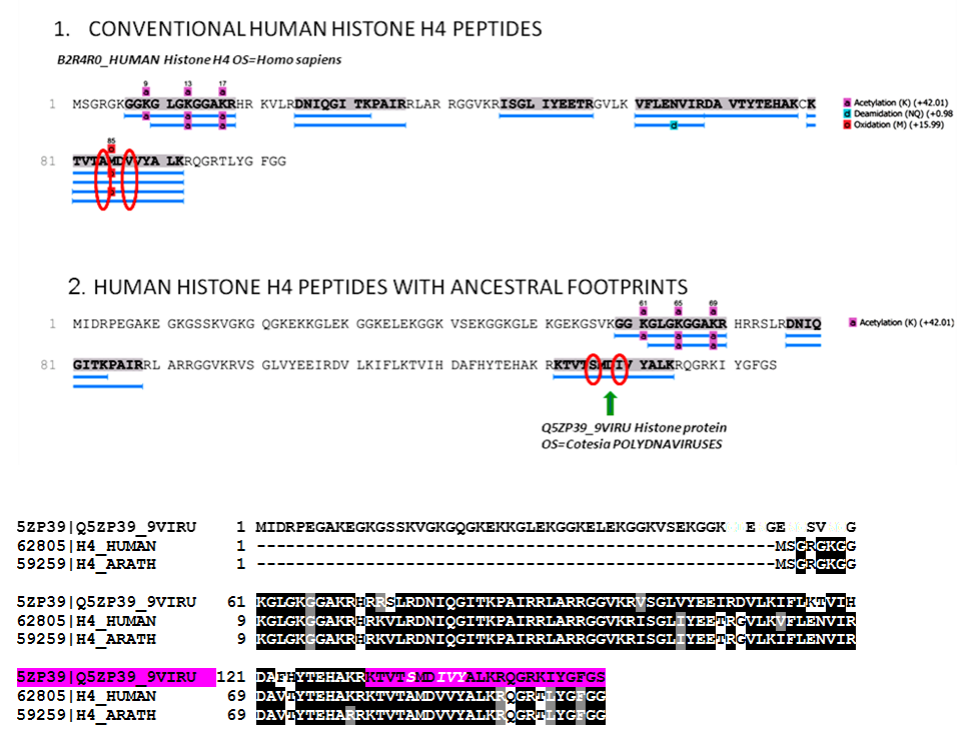
PEAKS Coverage view of the histone H4 peptides searched against the combined human-virus database, 1% of FDR. Section 1: The identified peptides are the canonical human histone-4 isoforms. Section 2: The histone H4 peptide KTV SMD IVYALK, indicated by the green arrow, has a unique footprint not found in any human or other eukaryotes proteins. This peptide displays the same unique sequence found in the C-terminus of the histone H4 of giant polydnaviruses (purple colour in the alligmnements between eukaryotes and polydnaviruses histone H4 sequences).

**Figure 12.**
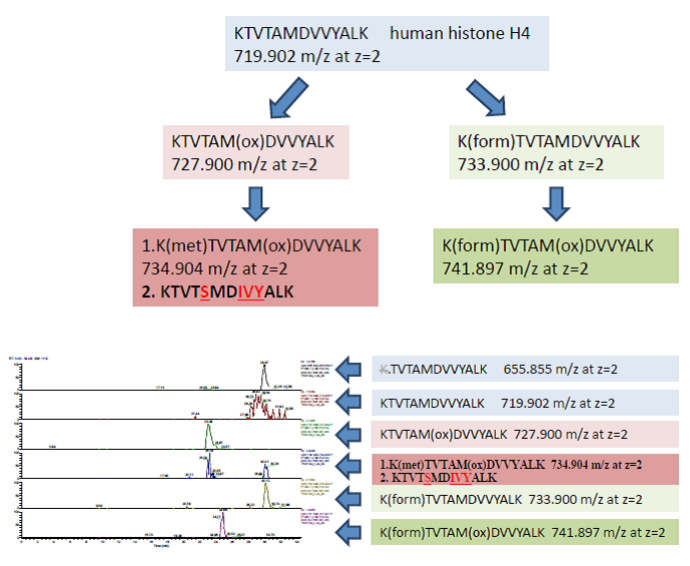
Proteomics data summary. Mass spectrometry identified human conventional and ancestral human H4 histone variants. Direct analyses and narrow scan in mass range 730–740 m/z; MSMS for center mass 734.40 m/z (2 ^nd^ isotope of 733.9 m/z; z=2); MSMS for center mass 744.40 m/z (2 ^nd^ isotope of 734.9 m/z; z=2) confirmed the co-existence of the human and ancestral H4 isoforms (with the IVY pattern) at 734.905 m/z; z=2.

Three dimensional (3D) protein models of the canonical human histone H4 protein and the histone H4 isoform having the viral footprint were generated by using the Swiss Model ( https://swissmodel.expasy.org/).

Entire project-raw mass spectrometry data of the positive protein spots (band 1–4) Click here for additional data file. Copyright: © 2017 Lusi EA et al. 2017 Data associated with the article are available under the terms of the Creative Commons Zero "No rights reserved" data waiver (CC0 1.0 Public domain dedication).

## Discussion

Although there are morphological and biochemical properties similar to giant viruses, the newly identified structures are possibly beyond the concept of typical viruses. The structures are ubiquitous in human tissues and are not associated to a specific medical disease. We are aware that being ubiquitous does not necessarily mean that these structures are not viruses and not being infectious does not imply that they are not viruses, since viruses can be also ubiquitous and not pathogenic ^11– 13^. However, the type of the histological pattern and the mass spectrometry identification do not completely rule out that these structures could be human cellular components having a viral footprint ^14– 16^. Like mitochondria that were originally bacteria cells and still retain the bacterial features ^17– 19^, the human Mimivirus-like structures manifest an ancestral origin. Some of the histone variants detected within the human structures have the same universal motifs associated to the same function that are also used by giant polydnaviruses to manipulate their host transcription. The ***IVY*** histone pattern, that is present in these structures, tells the cells that some genes should be "off".

**Figure 13.**
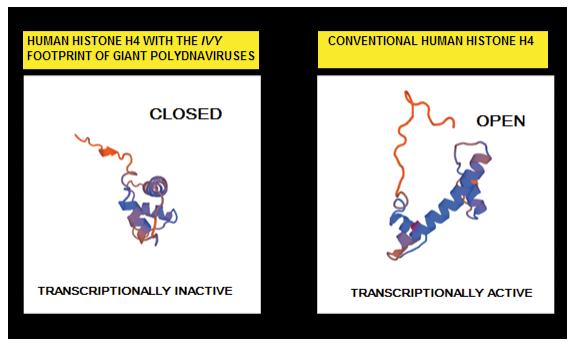
The newly identified human cellular structures display both canonical and histone H4 with an ancestral viral footprint. 3D protein structure models generated by SWISS-MODEL suggests that the human Mimivirus-like structures have a role in the regulation of transcription. The histone H4 with the IVY pattern have a closed transcriptionally inactive conformation. The canonical human histone H4 have an open conformation that is transcriptionally active.

The basis for this assertion corresponds to the findings of an identical ***IVY*** pattern in giant viruses that represses host gene transcription ^20– 22^. In addition, the three-dimensional analysis of the histone H4 that displays the ***IVY*** sequence shows a closed conformation that might prevent gene transcription ( Figure 13). It would be interesting to trace if an evolutionary link may exist between these human cellular structures, giant viruses or archaea. The recent finding that giant viruses can integrate into modern eukaryotic genomes have motivated the fascinating and highly provocative idea that giant viruses, along with archaea and bacteria, contributed significantly to the evolution of the first eukaryotes ^23– 26^.

## Conclusions

In conclusion, did we find ubiquitous giant viruses suppressing human responses or human structures with "something that was originally giant" and are not viruses any longer? The ancestral non-human nature of these structures is supported by the ***IVY*** histone pattern identified with mass spectrometry and by their capability to retain the Gram stain, which colour peptidoglycans. However, there are other alternative explanations for the structures that need to be considered as well. For example, the documented mammalian retroviral antigenicity does not entirely exclude the possibility that these structures could represent particles formed by the concurrent activity of retro-transposons.

By the virtue of development of the science of microscopy, the ultrastructure of the cell apparatus has been established by the 1960. Since then, new structures have been sporadically reported. The main challenge when uncovering cellular components is proteomics, which can be technically much more complex than transcriptomics, and electron microscopy is perceived by some scientists as an old fashioned technique prone to artefacts. However, it is worth mentioning that the Golgi apparatus was discovered with the use of a rudimental microscope in 1898 and many scientists did not believe that the Golgi apparatus was real and instead argued that the apparent body was a visual distortion caused by staining ^27– 30^. It took almost a century to fully understand the function of the Golgi apparatus. The aim of this paper is to merely report what we have found inside the human cells and offer some hypotheses. Only time and additional experiments will clarify if the identified structures are giant viruses having a retroviral antigenicity or cellular components having a viral ancestry or human retrotransposon-like elements.

## Data availability

The data referenced by this article are under copyright with the following copyright statement: Copyright: © 2017 Lusi EA et al.

Data associated with the article are available under the terms of the Creative Commons Zero "No rights reserved" data waiver (CC0 1.0 Public domain dedication). 

All the histological samples, slides, EM grids are available to be examined; please contact the corresponding author.

**Dataset 1: Entire project-raw mass spectrometry data of the positive protein spots (band 1–4).** doi, 10.5256/f1000research.11007.d153802 ^31^.

## Supplementary material

Supplemental File 1: FASTA file containing all identified proteins in the virus database and all identified proteins in the human database.

.

Supplemental File 2: Mass data and spectra of the identified histone H4 proteins.
