# Supplementary material for: Questions on unusual Mimivirus-like structures observed in human cells
Source: F1000Res. 2017 Mar 14;6:262. [Version 1] doi: 10.12688/f1000research.11007.1 (PMC5473404; doi:10.12688/f1000research.11007.1)
Supplement: Supplementary file 3 [file f1000research-6-11867-s0002.tgz › fd86605e-cd91-4fb1-ba48-4cd15b10db9a.docx]

**MS-data Narrow scan in the mass range 730-740 m/z; MSMS for center mass 734.40 m/z (2^nd^ isotope of 733.9 m/z; z=2); MSMS for center mass 744.40 m/z (2^nd^ isotope of 734.9 m/z; z=2)**

1. **KTVTAMDVVYALK** canonical H4 Histone MW = 1465.784

with formylation 733.900 m/z; z=2

489.602 m/z; z=3

**2. KTVTSMDIVYALK** Ancestral H4 Histone MW = 1467.801

734.908 m/z; z=2

490.274 m/z; z=3

***Ion chromatograms. Top🡪 bottom: Total ion chromatogram (TIC); extracted ion chromatogram (EIC) DB-hit; EIC H4 histone variant.***

***Mass spectrum at RT = 30.1 min***

***Zoom of mass spectrum at RT = 30.1 min***

***Mass spectrum at RT = 23.1 min***

***Zoom of mass spectrum at RT = 23.1 min***

***Secondary zoom of mass spectrum at RT = 23.1 min***

***top: original; bottom: delta-mass***

- ***Delta mass 7.0078; z=2 🡪 14.0156 = methylation***
- ***734.90 is probably methylated form of 727.90 m/z; z=2***

***DB-ID for 727.90 m/z; z=2 🡪 KTVTAM(ox)DVVYALK***

**Canonical human histone H4 and fragmentation table**

| **#** | **b** | **b++** | **b++-H2O** | **b++-NH3** | **b-H2O** | **b-NH3** | **Seq.** | **y** | **y++** | **y++-H2O** | **y++-NH3** | **y-H2O** | **y-NH3** | **#** |
| --- | --- | --- | --- | --- | --- | --- | --- | --- | --- | --- | --- | --- | --- | --- |
| **1** |  |  |  |  |  |  | **K** | **1454.792** | **727.900** | **718.894** | **719.387** | **1436.782** | **1437.766** | **13** |
| **2** | **230.150** | **115.579** | **106.574** | **107.066** | **212.140** | **213.124** | **T** | **1326.697** | **663.852** | **654.847** | **655.339** | **1308.686** | **1309.670** | **12** |
| **3** | **329.219** | **165.113** | **156.108** | **156.600** | **311.208** | **312.192** | **V** | **1225.649** | **613.328** | **604.323** | **604.815** | **1207.638** | **1208.623** | **11** |
| **4** | **430.267** | **215.637** | **206.632** | **207.124** | **412.256** | **413.240** | **T** | **1126.581** | **563.794** | **554.789** | **555.281** | **1108.570** | **1109.554** | **10** |
| **5** | **501.304** | **251.155** | **242.150** | **242.642** | **483.293** | **484.277** | **A** | **1025.533** | **513.270** | **504.265** | **504.757** | **1007.522** | **1008.506** | **9** |
| **6** | **648.339** | **324.673** | **315.668** | **316.160** | **630.328** | **631.313** | **M(ox)** | **954.496** | **477.752** | **468.746** | **469.238** | **936.485** | **937.469** | **8** |
| **7** | **763.366** | **382.187** | **373.181** | **373.673** | **745.355** | **746.339** | **D** | **807.461** | **404.234** | **395.229** | **395.721** | **789.450** | **790.434** | **7** |
| **8** | **862.434** | **431.721** | **422.716** | **423.208** | **844.424** | **845.408** | **V** | **692.434** | **346.720** |  | **338.207** |  | **675.407** | **6** |
| **9** | **961.503** | **481.255** | **472.250** | **472.742** | **943.492** | **944.476** | **V** | **593.365** | **297.186** |  | **288.673** |  | **576.339** | **5** |
| **10** | **1124.566** | **562.787** | **553.781** | **554.273** | **1106.556** | **1107.540** | **Y** | **494.297** | **247.652** |  | **239.139** |  | **477.270** | **4** |
| **11** | **1195.603** | **598.305** | **589.300** | **589.792** | **1177.593** | **1178.577** | **A** | **331.233** | **166.120** |  | **157.607** |  | **314.207** | **3** |
| **12** | **1308.687** | **654.847** | **645.842** | **646.334** | **1290.677** | **1291.661** | **L** | **260.196** | **130.602** |  | **122.089** |  | **243.170** | **2** |
| **13** |  |  |  |  |  |  | **K** | **147.112** | **74.060** |  | **65.546** |  | **130.086** | **1** |

KTVTAMDVVYALK human histone H4

719.902 m/z at z=2

K(form)TVTAMDVVYALK

733.900 m/z at z=2

KTVTAM(ox)DVVYALK

727.900 m/z at z=2

1.K(met)TVTAM(ox)DVVYALK

734.904 m/z at z=2

**2. KTVTSMDIVYALK**

KTVTAMDVVYALK 719.902 m/z at z=2

~~K~~.TVTAMDVVYALK 655.855 m/z at z=2

K(form)TVTAM(ox)DVVYALK

741.897 m/z at z=2

KTVTAM(ox)DVVYALK 727.900 m/z at z=2

**1.K(met)TVTAM(ox)DVVYALK 734.904 m/z at z=2**

**2. KTVTSMDIVYALK**

K(form)TVTAMDVVYALK 733.900 m/z at z=2

K(form)TVTAM(ox)DVVYALK 741.897 m/z at z=2

***~~K~~.TVTAMDVVYALK 655.855 m/z at z=2***

***KTVTAM(ox)DVVYALK 727.900 m/z***

***KTVTAM(ox)DVVYALK 727.900 m/z magnification view***

***MSMS of 734.904 m/z; z=2***

***MSMS of 734.904 m/z; z=2 magnification view***

**K(met)TVTAM(ox)DVVYALK m/z= 734.904; z=2 Score 2091.3 for MSMS delta of 0.5 Da**

| # | B | b++ | b++-H2O | b++-NH3 | b-H2O | b-NH3 | Seq. | y | y++ | y++-H2O | y++-NH3 | y-H2O | y-NH3 | # |
| --- | --- | --- | --- | --- | --- | --- | --- | --- | --- | --- | --- | --- | --- | --- |
| 1 |  |  |  |  |  |  | K(met) | 1468.80 | 734.90 | **725.90** | **726.39** | 1450.79 | 1451.78 | 13 |
| 2 | **244.16** | 122.58 | 113.58 | 114.07 | **226.15** | 227.13 | T | **1326.70** | **663.85** | **654.85** | **655.34** | **1308.69** | **1309.67** | 12 |
| 3 | **343.23** | 172.12 | 163.11 | 163.60 | 325.22 | 326.20 | V | **1225.65** | **613.33** | **604.32** | **604.81** | **1207.64** | 1208.62 | 11 |
| 4 | **444.28** | 222.64 | 213.64 | 214.13 | **426.27** | 427.25 | T | **1126.58** | **563.79** | 554.79 | **555.28** | **1108.57** | 1109.55 | 10 |
| 5 | **515.31** | 258.16 | 249.16 | **249.65** | **497.30** | 498.29 | A | **1025.53** | 513.27 | 504.26 | **504.76** | 1007.52 | 1008.51 | 9 |
| 6 | **662.35** | **331.68** | 322.67 | 323.16 | **644.34** | **645.32** | M(ox) | **954.50** | **477.75** | 468.75 | 469.24 | 936.49 | 937.47 | 8 |
| 7 | **777.38** | **389.19** | **380.19** | **380.68** | **759.37** | **760.35** | D | **807.46** | 404.23 | **395.23** | **395.72** | 789.45 | **790.43** | 7 |
| 8 | **876.44** | **438.73** | **429.72** | **430.21** | **858.43** | **859.42** | V | **692.43** | 346.72 |  | 338.21 |  | **675.41** | 6 |
| 9 | **975.51** | **488.26** | 479.25 | 479.75 | **957.50** | **958.49** | V | **593.37** | **297.19** |  | 288.67 |  | **576.34** | 5 |
| 10 | **1138.58** | **569.79** | **560.79** | **561.28** | 1120.57 | **1121.55** | Y | **494.30** | 247.65 |  | 239.14 |  | **477.27** | 4 |
| 11 | **1209.61** | **605.31** | **596.30** | **596.80** | **1191.60** | **1192.59** | A | **331.23** | 166.12 |  | 157.61 |  | 314.21 | 3 |
| 12 | **1322.70** | **661.85** | **652.85** | **653.34** | **1304.69** | **1305.67** | L | **260.20** | 130.60 |  | 122.09 |  | 243.17 | 2 |
| 13 |  |  |  |  |  |  | K | 147.11 | 74.06 |  | 65.55 |  | 130.09 | 1 |

**KTVTSMDIVYALK** m/z= 734.907; z=2 Score 1213.9 for MSMS delta of 0.5 Da

| # | B | b++ | b++-H2O | b++-NH3 | b-H2O | b-NH3 | Seq. | y | y++ | y++-H2O | y++-NH3 | y-H2O | y-NH3 | # |
| --- | --- | --- | --- | --- | --- | --- | --- | --- | --- | --- | --- | --- | --- | --- |
| 1 |  |  |  |  |  |  | K | 1468.81 | 734.91 | **725.90** | **726.39** | 1450.80 | 1451.78 | 13 |
| 2 | 230.15 | 115.58 | 106.57 | 107.07 | 212.14 | 213.12 | T | **1340.71** | **670.86** | 661.85 | **662.35** | **1322.70** | **1323.69** | 12 |
| 3 | 329.22 | 165.11 | 156.11 | 156.60 | 311.21 | 312.19 | V | **1239.66** | **620.34** | **611.33** | **611.82** | 1221.65 | 1222.64 | 11 |
| 4 | **430.27** | 215.64 | **206.63** | **207.12** | 412.26 | 413.24 | T | **1140.60** | **570.80** | **561.80** | **562.29** | 1122.59 | 1123.57 | 10 |
| 5 | **517.30** | 259.15 | **250.15** | 250.64 | **499.29** | 500.27 | S | **1039.55** | **520.28** | **511.27** | **511.76** | 1021.54 | 1022.52 | 9 |
| 6 | **648.34** | 324.67 | 315.67 | 316.16 | 630.33 | 631.31 | M | **952.52** | **476.76** | 467.76 | 468.25 | **934.51** | 935.49 | 8 |
| 7 | 763.37 | 382.19 | **373.18** | 373.67 | 745.36 | 746.34 | D | **821.48** | 411.24 | 402.24 | 402.73 | 803.47 | 804.45 | 7 |
| 8 | **876.45** | **438.73** | **429.72** | **430.22** | **858.44** | **859.42** | **I** | **706.45** | **353.73** |  | **345.21** |  | **689.42** | 6 |
| 9 | **975.52** | **488.26** | 479.26 | 479.75 | **957.51** | **958.49** | **V** | **593.37** | **297.19** |  | 288.67 |  | **576.34** | 5 |
| 10 | **1138.58** | **569.79** | **560.79** | **561.28** | 1120.57 | **1121.56** | **Y** | **494.30** | 247.65 |  | 239.14 |  | **477.27** | 4 |
| 11 | **1209.62** | **605.31** | **596.31** | **596.80** | **1191.61** | **1192.59** | **A** | **331.23** | 166.12 |  | 157.61 |  | 314.21 | 3 |
| 12 | **1322.70** | **661.86** | **652.85** | **653.34** | **1304.69** | **1305.68** | **L** | **260.20** | 130.60 |  | 122.09 |  | 243.17 | 2 |
| 13 |  |  |  |  |  |  | **K** | 147.11 | 74.06 |  | 65.55 |  | 130.09 | 1 |

**K(met)TVTSMDVVYALK m/z = 734.904; z=2 Score 1634.9 for MSMS delta of 0.5 Da**

| # | b | b++ | b++-H2O | b++-NH3 | b-H2O | b-NH3 | Seq. | y | y++ | y++-H2O | y++-NH3 | y-H2O | y-NH3 | # |
| --- | --- | --- | --- | --- | --- | --- | --- | --- | --- | --- | --- | --- | --- | --- |
| 1 |  |  |  |  |  |  | K(met) | 1468.80 | 734.90 | **725.90** | **726.39** | 1450.79 | 1451.78 | 13 |
| 2 | **244.16** | 122.58 | 113.58 | 114.07 | **226.15** | 227.13 | T | **1326.70** | **663.85** | **654.85** | **655.34** | **1308.69** | **1309.67** | 12 |
| 3 | **343.23** | 172.12 | 163.11 | 163.60 | 325.22 | 326.20 | V | **1225.65** | **613.33** | **604.32** | **604.81** | **1207.64** | 1208.62 | 11 |
| 4 | **444.28** | 222.64 | 213.64 | 214.13 | **426.27** | 427.25 | T | **1126.58** | **563.79** | 554.79 | **555.28** | **1108.57** | 1109.55 | 10 |
| 5 | **531.31** | 266.16 | 257.15 | 257.64 | 513.30 | 514.28 | S | **1025.53** | 513.27 | 504.26 | **504.76** | 1007.52 | 1008.51 | 9 |
| 6 | **662.35** | **331.68** | 322.67 | 323.16 | **644.34** | **645.32** | M | 938.50 | 469.75 | 460.75 | 461.24 | 920.49 | 921.47 | 8 |
| 7 | **777.38** | **389.19** | **380.19** | **380.68** | **759.37** | **760.35** | D | **807.46** | 404.23 | **395.23** | **395.72** | 789.45 | **790.43** | 7 |
| 8 | **876.44** | **438.73** | **429.72** | **430.21** | **858.43** | **859.42** | V | **692.43** | 346.72 |  | 338.21 |  | **675.41** | 6 |
| 9 | **975.51** | **488.26** | 479.25 | 479.75 | **957.50** | **958.49** | V | **593.37** | **297.19** |  | 288.67 |  | **576.34** | 5 |
| 10 | **1138.58** | **569.79** | **560.79** | **561.28** | 1120.57 | **1121.55** | Y | **494.30** | 247.65 |  | 239.14 |  | **477.27** | 4 |
| 11 | **1209.61** | **605.31** | **596.30** | **596.80** | **1191.60** | **1192.59** | A | **331.23** | 166.12 |  | 157.61 |  | 314.21 | 3 |
| 12 | **1322.70** | **661.85** | **652.85** | **653.34** | **1304.69** | **1305.67** | L | **260.20** | 130.60 |  | 122.09 |  | 243.17 | 2 |
| 13 |  |  |  |  |  |  | K | 147.11 | 74.06 |  | 65.55 |  | 130.09 | 1 |

***K(form)TVTAMDVVYALK 733.900 m/z***

***K(form)TVTAM(ox)DVVYALK 741.897 m/z at z=2***

**Bull & Breese index**

**TVTAMDVVYALK -3120 rank 3**

**KTVTAMDVVYALK -2660 rank 4**

**TVTSMDIVYALK -4010 highest hydrophobicity = rank 1**

**KTVTSMDIVYALK -3550 rank 2**
