# Supplementary material for: Questions on unusual Mimivirus-like structures observed in human cells
Source: F1000Res. 2017 Mar 14;6:262. [Version 1] doi: 10.12688/f1000research.11007.1 (PMC5473404; doi:10.12688/f1000research.11007.1)
Supplement: Peer review history

# Peer review: Referee response for version 1

**DOI:** 10.5256/f1000research.11867.r21292

**Published:** 2017-06-12

**Authors:** Didier Raoult

**Affiliations:** 1 Institut Hospitalo-Universitaire Méditerranée-Infection, Faculté de médecine, Aix-Marseille Université, URMITE, UM63, CNRS7278, IRD198, Inserm 1095, Marseille, France

Accept as it is

 This is a fascinating paper.

 This reviewer doesn’t know what it means anyway. However, Marseillevirus was reported in the blood of healthy donors that fuel the hypothesis of an asymptomatic carriage of giant viruses. Wholes sequencing of the positive samples after depletion of human genes may reveal if there is viral DNA.

I have read this submission. I believe that I have an appropriate level of expertise to confirm that it is of an acceptable scientific standard.

# Peer review: Referee response for version 1

**DOI:** 10.5256/f1000research.11867.r20949

**Published:** 2017-05-25

**Authors:** Carlo Presutti, Milena Grossi

**Affiliations:** 1 Dep. Biology and Biotechnology, University of Rome, Rome, 00185, Italy; 2 Istituto Pasteur Italia-Fondazione Cenci Bolognetti, Dipartimento di Biologia e Biotecnologie, Sapienza Università di Roma, Rome, Italy

The paper by Lusi *et al*. describes the identification of giant virus particles in human cells that seem to be quite ubiquitously and not related to any pathology. As long as I can judge, considering the fact that I am not a virologist nor an EM (electron microscopy) expert, the experiments seem to be clear and well executed. In particular the mass spectrometry experiments sound convincing.

 Authors indicate that "Mimivirus-like structures identified in the human cells were ubiquitous and manifested a distinct mammalian retroviral antigenicity." However no data or experiments are shown about this interesting feature. This could be included in the paper or at least added to the discussion. The authors should also indicate why they did not go for nucleic acid identification: this could be an easier and clearer way to characterize these organisms.

  The Gram-positive staining of human tissues described by the Authors is quite curious and potentially interesting, although the images presented are not so clear. Moreover, same magnification should be shown for all samples in order to better appreciate the differences among different tissues underlined by the Authors. Concerning the EM micrograph, while the virus particles inside the amoeba cell are clearly visible, is the giant particle in liver cells that indicated by the two arrowheads? If so, the similarities that can be appreciated are just the large dimensions, as they appear rather different in morphology. The Authors also refer to a retroviral antigenicity associated to the granules, as determined by staining with anti p27-gag. In the MM of the manuscript a western blot for this protein is also described, however I could not find any data about them. Since the antibody used was specific for the p27-gag from FeLV, was the cross-reactivity with the human retrovirus Gag protein tested?

 The data from mass spectrometry analysis look interesting, although it is not clear to me what kind of tissues were analysed.

 Albeit the data would need to be improved as suggested, the findings reported appear very intriguing and of interest for future developments. Certainly, it seems strange that no one has ever appreciated the presence of these intracellular structures before.

We have read this submission. We believe that we have an appropriate level of expertise to confirm that it is of an acceptable scientific standard.
